# The COACH prompting system to assist older adults with dementia through handwashing: An efficacy study

**DOI:** 10.1186/1471-2318-8-28

**Published:** 2008-11-07

**Authors:** Alex Mihailidis, Jennifer N Boger, Tammy Craig, Jesse Hoey

**Affiliations:** 1 Department of Occupational Science and Occupational Therapy, University of Toronto, 160-500 University Ave, Toronto, ON, M5G 1V7, Canada; 2 Toronto Rehabilitation Institute, Toronto, ON, Canada; 3 School of Computing, University of Dundee, Dundee, UK

## Abstract

**Background:**

Many older adults with dementia require constant assistance from a caregiver when completing activities of daily living (ADL). This study examines the efficacy of a computerized device intended to assist people with dementia through ADL, while reducing caregiver burden. The device, called COACH, uses artificial intelligence to autonomously guide an older adult with dementia through the ADL using audio and/or audio-video prompts.

**Methods:**

Six older adults with moderate-to-severe dementia participated in this study. Handwashing was chosen as the target ADL. A single subject research design was used with two alternating baseline (COACH not used) and intervention (COACH used) phases. The data were analyzed to investigate the impact of COACH on the participants' independence and caregiver burden as well as COACH's overall performance for the activity of handwashing.

**Results:**

Participants with moderate-level dementia were able to complete an average of 11% more handwashing steps independently and required 60% fewer interactions with a human caregiver when COACH was in use. Four of the participants achieved complete or very close to complete independence. Interestingly, participants' MMSE scores did not appear to robustly coincide with handwashing performance and/or responsiveness to COACH; other idiosyncrasies of each individual seem to play a stronger role. While the majority (78%) of COACH's actions were considered clinically correct, areas for improvement were identified.

**Conclusion:**

The COACH system shows promise as a tool to help support older adults with moderate-levels of dementia and their caregivers. These findings reinforce the need for flexibility and dynamic personalization in devices designed to assist older adults with dementia. After addressing identified improvements, the authors plan to run clinical trials with a sample of community-dwelling older adults and caregivers.

## Background

Globally, the number of individuals aged 65 years and older is predicted to increase steadily, particularly among the oldest old (aged 80 years and over) after the year 2010 [1]. This will result in an increase in the worldwide number of individuals diagnosed with dementia, particularly Alzheimer's Disease (AD), from the current estimate of 24.3 million individuals in 2006 to 81.1 million by 2040 [2].

Older adults have a strong preference for aging-in-place (i.e. remaining in their own homes and communities) compared to other forms of care, such as nursing homes and other long-term care facilities [3]. Additionally, various studies have implied that older adults (particularly those who have AD) benefit from aging in environments to which they are accustomed as familiar environments can provide memory and task cues [4-6]. However, this shift from the hospital to home-based care means that family members and other informal caregivers are being increasingly depended upon to attend to the long-term health-care needs of older adults with AD. Increased dependence and changes in the relationship dynamic are difficult for both people with AD and their family caregivers to accept [7]. The constant pressure to meet their relative's needs for assistance and support can result in debilitating levels of stress for the caregiver, resulting in the affected person's placement into long-term care. From a caregiver's perspective, decreasing the number of interactions required to complete an activity of daily living (ADL) has a direct positive impact on caregiver burden. Even small decreases in caregiver burden have been found to alleviate the prevalence of depressive symptoms in caregivers of individuals with AD [8]. This can lead to more successful informal care, resulting in lower medical costs and delayed long-term care placements.

To support aging-in-place, older adults and their caregivers are increasingly relying on the use of computerized *Cognitive Assistive Technologies (CATs) *to complete ADL [6]. Often coupled with some form of artificial intelligence (AI), CATs strive to support cognitive disorders thereby enhancing the user's autonomy [9]. The maintenance or increase of independence is coupled with a reduction in the levels of caregiver assistance, and likely caregiver burden, as well as a decrease in home heath care costs [10].

A significant amount of recent work in CATs for assisting people with cognitive impairments use probabilistic models to infer task and occupant status from sensors distributed throughout a person's living environment [11]. For example, Autominder, developed by Pollack et al. [12], uses artificial intelligence planning to schedule events such as medication taking around a person's daily schedule, such as favorite television programs or daily walks. Autominder uses environmental sensors to detect the status of activities, and if required, will provide the user with context-aware reminders regarding unattended activities. The Gator Tech Smart House is an example of a smart home designed with older adults in mind. Sensors distributed throughout the house interact with applications running on computers to take into account context when performing actions. For example, if it is a sunny day outside and the resident has the television on, the Gator Tech Smart House [13] will automatically close the blinds to reduce glare. Other features include medication reminders that can appear on the bathroom mirror and automatic sensing and ordering for soap and toilet paper refills. Pigot et al. [14] developed Archipel, a cognitive modeling system for cooking tasks that recognizes the user's intended plan and adapts prompting to a pre-determined cognitive impairment level. Sensors, such as RFID tags and readers, in the kitchen environment detect which objects have been used and provide cues (audio, video and strategic lighting) to help users through each step in the task. As with Autominder, Archipel will not give reminders for tasks the user has already accomplished.

Research is increasingly emphasizing the importance of maintaining functional independence in older adults as a way of maintaining good health and wellness among older adults with dementia, while simultaneously reducing medical expenditures [15,16]. However, the extent to which CATs can aid an individual with AD depends on the users' willingness to implement it, which in turn depends on whether the individual and/or his/her caregiver can operate the device, feels that the device is useful, and whether the device supports or undermines the sense of personal identity [17]. To be useful to both a care recipient with dementia and his/her caregiver(s), a CAT must be autonomous, non-invasive, and must not require explicit feedback (e.g. button presses), as this cannot reasonably be expected of either people with AD or overworked caregivers. Cognitive assistance should be able to accommodate high levels of customization as the more the assistance is personalized and appropriate to the deficits in question, the more likely it will be adhered to and understood by the user [18]. Finally, assistance should only be given on an "as needed" basis to minimize confusion and to keep the user as cognitively involved in the task as possible.

The majority of currently available CATs require extensive sensor deployment and maintenance and/or input from a cognitively intact individual. Most likely the caregiver of the individual with dementia would have to learn how to operate and (to some degree) maintain a potentially complex planning system. As many caregivers are overburdened as it is, two goals of the system described in this paper were to minimize the amount of hardware that was needed, and to have the system function without any explicit input from the user or the caregiver.

The result was the COACH (**C**ognitive **O**rthosis for **A**ssisting a**C**tivities in the **H**ome), a system that employs various computer vision and artificial intelligence techniques to autonomously provide the user with verbal and/or visual reminders as necessary during ADL. Table 1 summarizes the progression of the systems used in the previous versions of COACH. The systems in each version of COACH represent significant advances in the sophistication and versatility compared to those used in the previous version. The systems for the latest version of COACH (Version 3 in Table 1) are described in more detail in the Methods section below.

**Table 1:** Summary of previous COACH systems.

**COACH Version**	**Tracking System**	**Decision-making system**	**Prompting system**	Number of Subjects*	**Related Publications**
Version 1	Pattern wristband worn by the user	Neural networks interacting with a hard-coded taxonomy	Audio prompts, with one prompt for each step	10	[22,33]
Version 2	Using background subtraction to isolate the user's hands. Tracking of hands and task objects (i.e. soap and towel) using a preset colour model.	Fully observable Markov decision process (MDP). This technique assumes the world is fully observable; it does not take into account hidden variables, such as user responsiveness.	Audio prompts with three levels of assistance (minimal, moderate, and maximal) for each step.	4	[20,37-39]
Version 3 (system presented in this paper)	Colour based flocking.	[Belief monitoring system & policy] Partially observable Markov decision process (POMDP). This model takes into account hidden variables and is able to make decisions in conditions of uncertainty.	Audio and audio-video prompts with three levels of assistance (minimal, maximal, and maximal + video demonstration) for each step. Encouragement and a reminder regarding the activity the user is attempting added. Professional actor recorded prompts.	6	[18,19,21,23,25]

* Results from previous trials are not presented as they are not comparable because of the variance in the technologies and procedures that were used.

This paper presents results from an eight-week efficacy study of the COACH with older adults with dementia. Methods and results are presented, followed by a discussion regarding the potential clinical significance of the participants' and device performances. While a brief description of the technology will be provided in this paper, the reader is referred to [19] for an in-depth description of the COACH system and algorithms.

## Objective

The objective of this study was to answer the following research questions:

1. Is the COACH system able to guide an older adult with dementia through the handwashing ADL with less dependence on a caregiver? If dependence decreases it should be reflected in an increase of the number of steps in the handwashing activity the older adult is able to complete independently from a caregiver (i.e. with no assistance from the caregiver).

2. Does this new version of COACH reduce caregiver workload? If the caregiver's workload is reduced, this should be reflected in a decrease in the number of times a caregiver interacts with his/her care recipient.

3. How will the COACH system perform with respect to its ability to correctly provide assistance to the user throughout the ADL? To achieve a positive outcome, the system must be able to follow the older adult through the handwashing task, autonomously giving the correct prompt if (and only if) they are needed.

## Methods

### Device (COACH) design

In this work the authors extend upon the two previous versions of the COACH device (summarized in Table 1), which both focused on the activity of handwashing [20-22]. Handwashing was chosen as the model ADL because it is a relatively safe activity that older adults with dementia have difficulties completing because of the required planning and initiation skills.

**Figure 1:**
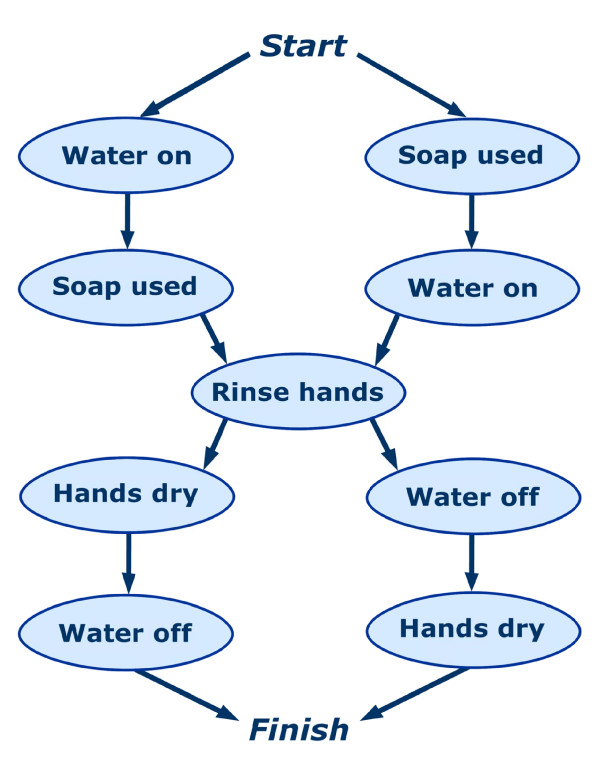
**The five essential steps of handwashing**. Successful activity completion was considered to be any sequence of steps that took the participant from "Start" to "Finish". As the long-term care facility's guidelines required the use of liquid soap wetting one's hands before getting the soap was not considered an essential step in the activity, therefore the water on and soap used steps are interchangeable.

**Figure 2:**
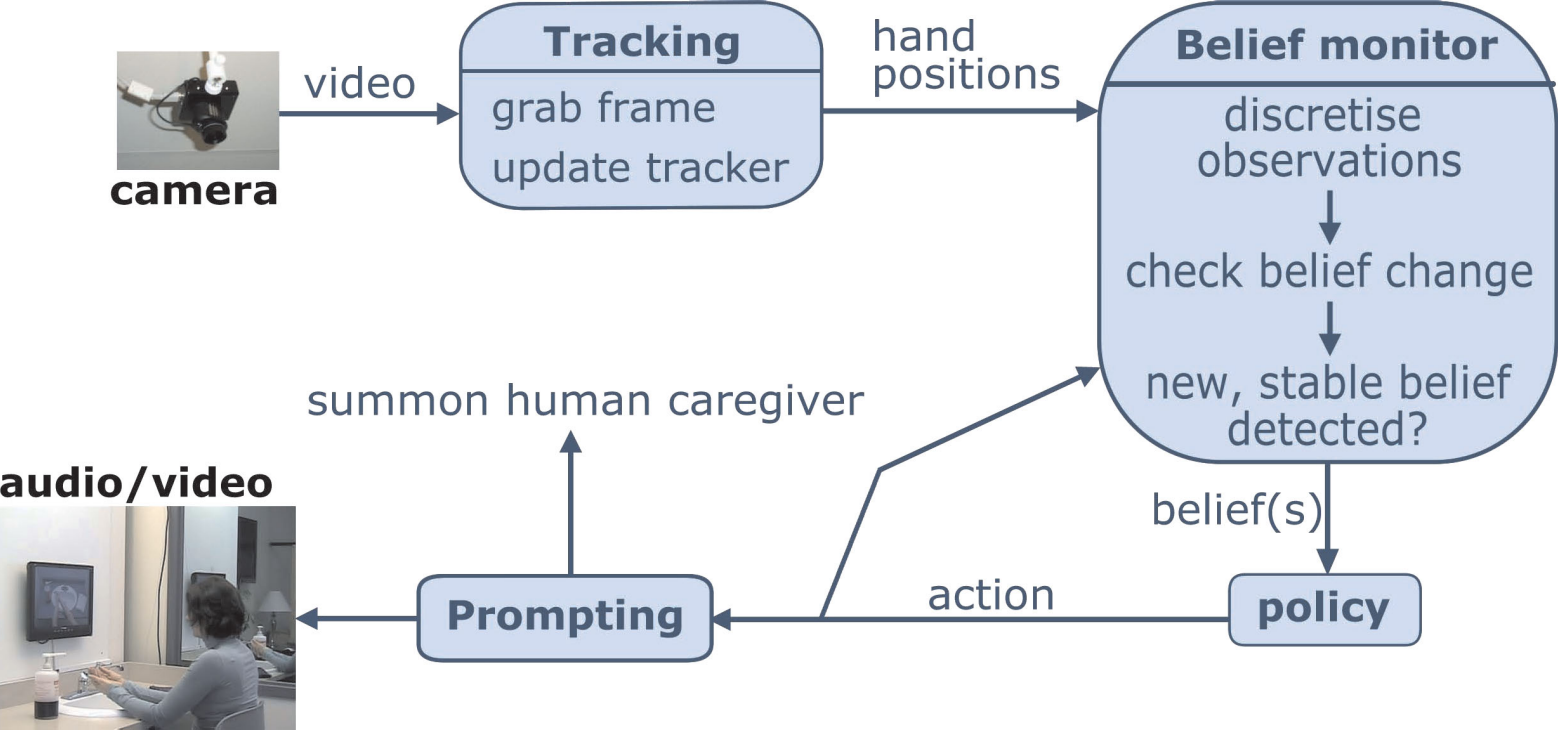
**A flow diagram of COACH components**. Images from the camera are translated into hand and towel positions by the tracking system. These are passed to the belief monitor, which calculates the probability distribution over the possible states. This belief state is passed to the policy, which selects an action for COACH to take (i.e. prompt, observe user, or call caregiver).

Handwashing was defined as having five essential steps that must be accomplished for successful activity completion, which are depicted in Figure 1. COACH guided users through these steps using four integrated components: the tracking system, belief monitoring system, policy, and prompting system, as represented in Figure 2.

Images captured by a video camera are processed by the tracking system and the hand and towel positions are passed to the belief monitoring system. These data are used by the belief monitoring system to compute the *belief state*; a probabilistic estimation of the current state of the user and environment. The belief state is passed from the belief monitoring system to the policy, which is essentially a lookup table that denotes the best course of action for the system to take for every state that could be received from the belief monitor. Each belief state that is received from the belief monitoring system is translated by the policy into an action for COACH to take. Possible actions available to the COACH are to give a low-guidance verbal prompt, give a high-guidance verbal prompt, give an verbal prompt with a video demonstration of the action, call the caregiver to intervene, or to do nothing (i.e., continue to observe the user). The COACH's different levels of prompting assistance give COACH the ability to select the most appropriate support for each individuals' stage of AD and overall responsiveness. Thus the level of detail played for the user is based on factors such as the error committed, sensory and cognitive status of the user, and past responsiveness to the previous prompts.

The COACH system presented above had three significant changes from the previous versions: 1) The use of markerless flocking to track the activity; 2) the use of a *partially observable Markov decision process (POMDP) *to model the handwashing guidance problem; and 3) the refinement of audio prompts and the addition of video demonstrations. Tracking was accomplished using a computer vision technique known as flocking, which was developed by Hoey et al. [23]. It uses models of skin and towel color combined with a Bayesian sequential estimation technique. This method of tracking is quite robust and able to dependably track the location of the user's hands and the position of the towel, even after occlusion by an object or after leaving and returning to the camera's field of view. A POMDP was chosen as the basis for the new planning system because of this model's ability to make good decisions in situations of uncertainty, as well as making intelligent inferences, and therefore decisions, about unobservable states (e.g. a user's level of dementia) [24]. This type of model allows the COACH system to autonomously tailor itself to the individual needs of its users because it can estimate and use individual's traits (e.g. cognitive awareness and responsiveness levels) to dynamically adapt to daily and long term needs. Implementation of a POMDP is an important contribution to not only the field of artificial intelligence but to the usability concerns of users and their caregivers as it enables user-specific prompting strategies while remaining autonomous. Greater details regarding the technical nature of COACH, including system detailed descriptions and planning algorithms, can be found in [19].

**Figure 3:**
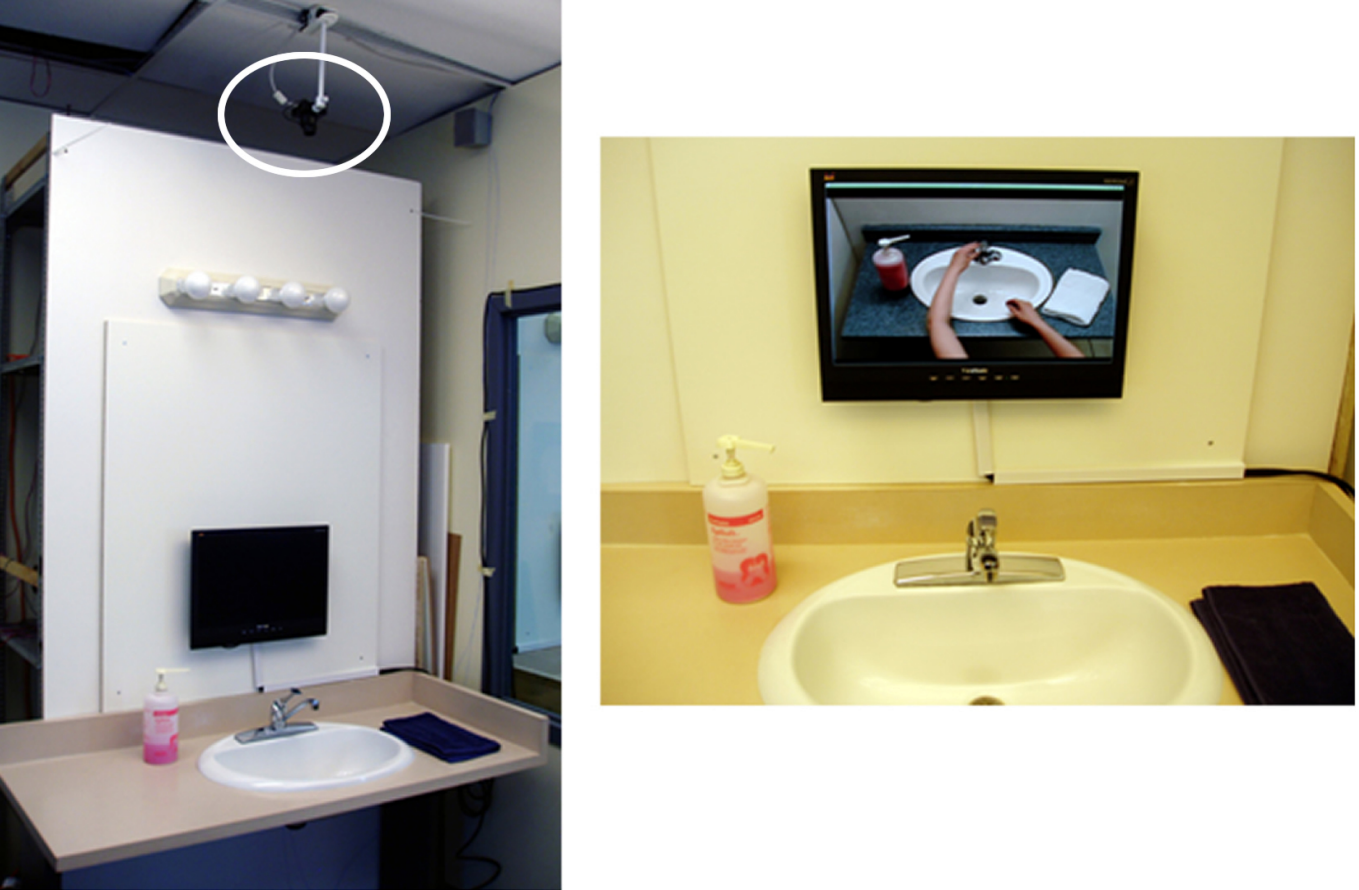
**Example of the COACH system setup**. (a) Example of the LCD screen, speakers (top-right), and video camera (circled) setup. (b) Example of a video-based prompt demonstrating how to turn on the water.

Audio prompts were recorded using a professional male actor to emulate the cadence and tone of a professional caregiver. A male voice was used (as opposed to a female one) because previous research by this group and others suggests that male voices are easier to hear and understand, possibly because the male voice has a lower pitch/frequency [22]. The wording used for the prompts is shown in Table 2 and was similar to the wording used in previous studies, modified slightly according to recommendations from Wilson et al. [18]. Prompts included the participant's name at the beginning of each prompt to get his/her attention. Previous studies with COACH have found that some users can get confused about which activity they were asked to complete (e.g. previous participants have been known to wash the towel in the sink, wash his/her face, etc.), therefore the prompt often contained a reminder to help participants remember which activity they were attempting to complete. The prompt then gave the participant guidance for the step in the activity s/he was attempting. The potential usefulness of adding video demonstrating correct completion of the activity step was examined by Labelle and Mihailidis [25]. Results were positive; therefore audio-video capabilities were added to this version of COACH. The videos used in this study were shot from the perspective of the participant. They were pre-recorded in the same washroom that was used in the trials and combined with the maximal assistance verbal prompts. A frame from one of the videos is shown on the monitor in Figure 3b.

**Table 2:** Wording for the prompts used by COACH.

**Step**	**Minimal verbal assistance**	Maximum verbal/video assistance*
Turn on the water	[Name], you're washing your hands. Can you turn the water on?	[Name], try turning the silver knobs.
Use the soap	[Name] you're washing your hands. Please use the soap.	[Name] you're washing your hands. Try putting on some soap.
Rinse hands	[Name], you're washing your hands. Please rinse your hands.	[Name], you're washing your hands. Try putting your hands in the water.
Turn off the water	[Name] you're doing great. Can you turn the water off?	[Name], you're doing great. Twist the knobs to turn the water off.
Dry hands	[Name], you're doing great. Dry your hands now.	[Name], try using the towel.

* The same wording was used for the maximum verbal assistance and video assistance prompts.

### Participants and Ethics/Consent Process

This study was reviewed and approved by the Toronto Rehabilitation Institute's Research Ethics Board (REB). Potential participants were identified by the staff at the long-term care (LTC) facility in Toronto, Canada where the study took place. Informed consent to participate was obtained in writing (using a the consent form approved by the REB) from the participants' substitute decision makers, after the study was described to them using an information sheet and informal interview.

Participants had to meet the following inclusion/exclusion criteria: over the age of 65 years of age, no history of violence, fluent in English, can hear normal levels of speech, exhibit no severe motor impairments, and have moderate-to-severe dementia. Level of dementia was determined through the administration of the Mini-Mental State Examination (MMSE), an assessment instrument that is commonly used to estimate the level of cognitive impairment in adults [26]. Typically, participants are separated into four categories of impairment based on his/her MMSE score: no impairment (30–26 points), mild (25–20 points), moderate (19–10 points), and severe (9-0 points). Each participant's dementia level was scored using the MMSE before the start and upon completion of the trials

### Apparatus set-up

The study was conducted in a retrofitted washroom and adjoining office that were dedicated to the project by the LTC facility. The washroom was fitted with a ceiling-mounted IEEE-1394 digital video camera (Point Grey Research DragonFly2), and a wall-mounted 21-inch LCD screen and desktop speakers (Figure 3a). A Dell Latitude laptop computer (2 GHz processor, 2 Gb RAM) was used as the processing unit for the system software and hardware, as well as the operator graphical user interface to display information about the system variables (e.g. estimated plan steps, system response, etc.), and the participant's progress through the task. The trials were also recorded using a camcorder positioned above the participant to capture video for post-trial evaluation by human raters (but was not used by the system during the trials).

### Study design

A single subject research design (SSRD) was used in this study because of the difficulty in recruiting and maintaining an adequate sample size and the variability of the participants' health [27-31]. This research design has been used in the authors' previous studies and has been found to be the most appropriate procedure for the evaluation of this type of technology. The study consisted of two baseline phases, A_1 _and A_2 _(COACH not used), and two intervention phases, B_1 _and B_2_, (COACH used), run in the order A_1_-B_1_-A_2_-B_2 _to identify any carry-over effects. Based on studies completed with previous versions of COACH, 10 trials per phase were deemed to be sufficient for participant performance to stabilize and for the desired changes in the dependent variables to be observed [22].

### Procedure

Trials consisted of one trial per day per participant, Mondays to Fridays, for eight weeks for a total of 40 trials each. To ensure uniformity and avoid any potential risk of injury from falls, each participant was required to sit in a wheelchair and was taken to the test washroom by a caregiver who was hired for this study. The caregiver positioned the participant in front of the sink in the test washroom and asked the participant to wash his/her hands.

During the A-phases of the trials, the caregiver interacted with the participants as she normally would, providing any prompts and reminders she felt were necessary to complete handwashing. During the B-phases, COACH was started by a researcher (who was hidden from the user) as soon as the caregiver requested the participant to wash his/her hands. The caregiver then left the participant alone in the test washroom and discreetly observed him/her from the hallway. The caregiver provided assistance only if instructed to do so by COACH (i.e., the caregiver was summoned by the device to intervene) or if the caregiver felt the need to intervene for the well being of the participant (e.g., the participant was attempting to stand up from the wheelchair or was becoming upset).

### Data collection tools

A score sheet was used to collect data required to evaluate the system's efficacy in terms of both user and system performance. The score sheet was the same one that was developed and used in studies that examined previous versions of the COACH (refer to [22]).

With respect to user performance, scales on the score sheet measured the following for each step of the activity: 1) independent step completion; 2) number of caregiver interactions; and 3) functional assessment scale (FAS). Independent step completion was scored for every trial. Participants scored one point for the first time s/he completed a step in a trial without assistance of any kind from a human caregiver. As there were five essential steps (Figure 1) the maximum score that could be attained was five, even if a participant completed more than five steps independently. For example, a participant could independently turned the water on, wet her hands, turned off the water, got some soap, turned the water on again, rinsed her hands, turned off the water, and finally dried her hands. Although none of the actions in this sequence would be technically incorrect, the participant would still score a five on independent step completion. Number of caregiver interactions was a count of the number of times the caregiver had to interact with the participant to get him/her to complete a step. An interaction was considered to be any exchange between the caregiver and the participant that was related to activity completion, including verbal prompting, demonstration, and touching (either the participant or an object). The functional assessment score (FAS) is a modified version of the Functional Independence Measure (FIM™), which is a standardized assessment tool used to measure one's ability to function with independence over 18 activities of daily living [32]. Participants received an FAS for each step in the activity and scores ranged from zero (no attempt/refusal) to seven (complete independence), with an overall maximum of 35. If the participant completed the step in response to prompts provided by the COACH, a score of seven was given. A higher cumulative FAS is expected to correlate with higher levels of activity completion independence. The face validity of the FAS was demonstrated in previous trials by Mihailidis et al. [22,33].

During the B-phases, data were collected regarding the system responses to participant performance during the handwashing activity. These data were collected based on the basic principles of signal detection theory (SDT) [34], which can be used to measure four conditions describing device performance with respect to: hits, false alarms, correct rejects, and misses. These conditions with respect to the COACH system are outlined in Table 3. For each step in the activity, the system was rated as having at least one, and potentially more, of the four possible SDT conditions. For example, if the COACH gave three incorrect prompts and one correct prompt for a step, three false alarms and one hit would be scored.

**Table 3:** The four possible conditions used to determine COACH's performance.

		**COACH Response**	
		
		***Prompt***	***No prompt***
**Participant Action**	***Error***	Hit	Miss
	
	***No error***	False Alarm	Correct Reject

### Analysis of participant and device performance

Video of each trial was reviewed and scored by an experienced rater using a multi-modal score sheet to collect the types of data described previously. An experienced rater was a researcher who was trained on the scoring methods and has had previous experience rating COACH trials. Space on each score sheet was provided for any general comments or observations.

Because of the small number of participants in the study, visual analyses of the data were used to identify trends of participant behaviors and abilities, and compare changes in variability between phases. Visual analysis is a commonly used technique for single-subject research designs. Data were examined for all trials and overall trends of in-group performance between baseline (A) and intervention (B) phases, as well as for variations in participant performance. Observed participant behaviors and reactions were used to aid in the analysis of the results.

Analyses of the device performance data were achieved through the calculation of the number of hits, misses, false alarms, and correct rejects (described in Table 3) made by the system during the intervention (B) phases. These data were also used to calculate two types of error: E_w _(Equation 1) which reflects COACH not detecting an error when participants made one, thus not giving a prompt, and E_c _(Equation 2) which reflects COACH detecting an error when none occurred, thus erroneously giving a prompt. These equations were derived by Mihailidis [33] for the analysis of previous research on COACH.

(1) $$E_{W}=\frac{Misses}{Hits+Misses}\times 100$$

(2) $$E_{C}=\frac{False\;Alarms}{False\;Alarms+Correct\;Rejects}\times 100$$

## Results

### Inter-rater agreement

To ensure data reliability, a second experienced rater scored 20 percent of all data collected regarding participant performance and an inter-rater agreement was calculated using Cohen's Kappa (using SPPS v15.0) [27]. The measures of agreement (*K *values) were K = 0.96 (p < 0.0005) for independent step completion, K = 0.69 (p < 0.0005) for number of caregiver interactions, and K = 0.63 (p < 0.0005) for FAS.

### Participants

Eight participants were recruited for this study, however two were withdrawn; S2 developed unrelated health problems, and S7's aggressive behavior caused concerns for the wellbeing of both herself and the study caregiver. Demographics for the remaining six participants are presented in Table 4. Based on his/her initial MMSE scores, five participants (S3, S4, S5, S6 and S8) were classified as having moderate-level dementia, and one participant (S1) was classified as having severe-level dementia.

**Table 4:** Demographics of the participants.

**Participant**	**Gender**	**Age (years)**	**Education**	**MMSE: study start**	**MMSE: study completion**	**Average MMSE**
S1	F	88	High school	5	3	4
S3	F	73	Post-secondary	12	18	15
S4	F	92	Elementary school	10	13	12
S5	M	81	Post-secondary	19	20	20
S6	F	87	High school	12	14	13
S8	F	89	Post-secondary	11	10	11

### Participant performance

**Figure 4:**
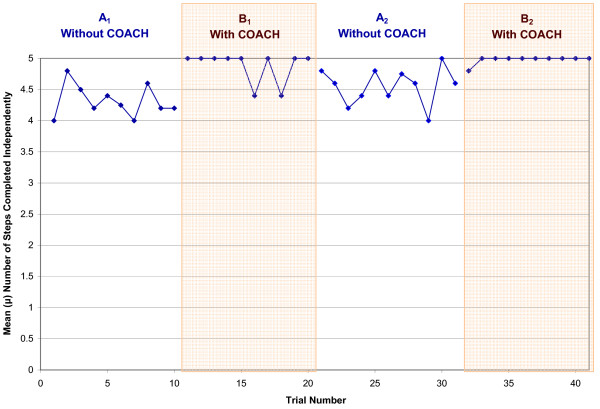
**Mean number of steps completed independently for all participants for each day of the trials**. A_1 _and A_2_are the baseline phases (no use of COACH), B_1_and B_2 _are the intervention phases (COACH used).

**Figure 5:**
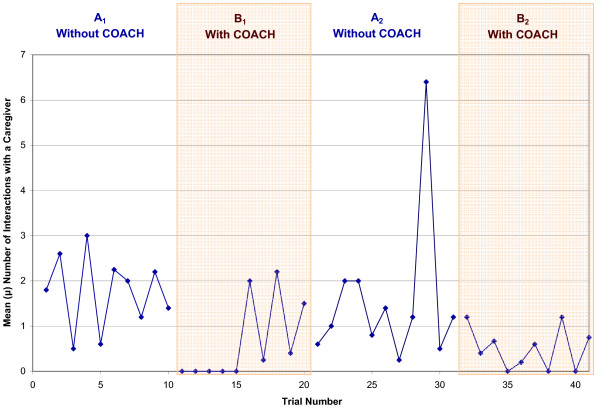
**Mean number of interactions with a human caregiver for all participants for each day of the trials**. A_1 _and A_2 _are the baseline phases (no use of COACH), B_1 _and B_2 _are the intervention phases (COACH used).

**Figure 6:**
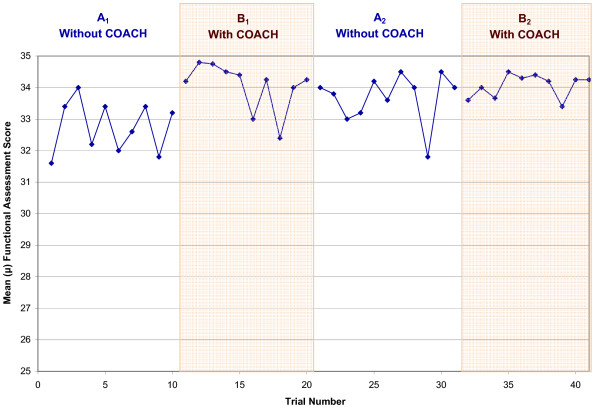
**Mean Functional Assessment Score (FAS) for all participants for each day of the trials**. A_1 _and A_2 _are the baseline phases (no use of COACH), B_1 _and B_2 _are the intervention phases (COACH used).

As S1 was the only participant in the severe-level group and noticeably different trends from the other participants, this sub-section examines the moderate-level participants (S3, S4, S5, S6 and S8) as a group. Table 5 summarizes overall individual participant performance per test phase, which shows improvements in all three areas, particularly in a reduction in the number of interactions with the caregiver. From Table 6 it can be seen that four of the five participants were able to independently complete the activity. Table 7 shows the overall number of interactions with the caregiver required by the participant to successfully complete essential handwashing steps, which decreased by an average of 66% when the device was introduced. Table 8 shows the participants' FAS for the handwashing activity increased by a negligible 2% for the group. Figures 4 to 6 depict the daily average performance for the entire moderate-level participant group (n = 5) for the number of steps completed independently, the number of interactions with a caregiver, and FAS respectively.

**Table 5:** Average participant performance for each trial phase and overall group performance.

**Participant [Average MMSE score]**	**Phase**	**Mean number of steps completed independently (out of 5)**	**Mean number of interactions with human caregiver**	Mean FAS* (out of 35)
Severe-level dementia**				
S1 [4]	A1	0.1	14.1	2.4
	B1	1.8	10.6	17.1
	A2	0.6	16.2	7.3
	B2	0.9	20.6	11.2
**Moderate-level dementia**				
S3 [15]	A1	5.0	0.0	35.0
	B1	5.0	0.0	35.0
	A2	4.9	0.2	34.8
	B2	5.0	0.0	35.0
S4 [12]	A1	3.6	2.6	29.9
	B1	5.0	0.0	34.7
	A2	4.5	1.4	33.9
	B2	5.0	0.0	34.5
S5 [20]	A1	3.3	4.4	30.7
	B1	4.1	2.6	31.6
	A2	3.8	3.8	32.2
	B2	4.9	0.3	33.2
S6 [13]	A1	5.0	0.0	35.0
	B1	5.0	0.0	34.9
	A2	5.0	0.3	34.8
	B2	5.0	0.0	34.9
S8 [11]	A1	4.6	1.9	33.2
	B1	5.0	1.3	33.1
	A2	4.5	2.2	32.8
	B2	5.0	2.6	32.3
Mean score over phases for the moderate-level group	A1&A2	4.4	1.7	33.2
	B1&B2	4.9	0.6	34.1
% change***		11	-66	2.4

Functional assessment score

** See Tables 6 to 8 for mean scores for S1

*** Calculated by [(B_1_+B_2_)-(A_1_+A_2_)]/(A_1_+A_2_)*100

**Table 6:** Average number of steps per trial completed independently without (Phase A) and with (Phase B) COACH

**Participant [MMSE]**	**Mean number of steps completed independently in Phase A (out of 5)**	**Mean number of steps completed independently in Phase B (out of 5)**	**Change (%)**
S1 [4]	0.38	1.33	250
S3 [15]	4.95	5.00	1
S4 [12]	4.10	5.00	22
S5 [20]	3.60	4.53	26
S6 [13]	5.00	5.00	0
S8 [11]	4.57	5.00	9

**Table 7:** Average number of interactions with the caregiver per trial without (Phase A) and with (Phase B) COACH

**Participant [MMSE]**	**Mean number of interactions in Phase A**	**Mean number of interactions in Phase B**	**Change (%)**
S1 [4]	15.19	15.56	2
S3 [15]	0.11	0.00	-100
S4 [12]	1.95	0.00	-100
S5 [20]	4.10	1.33	-68
S6 [13]	0.15	0.00	-100
S8 [11]	2.05	1.94	-5

**Table 8:** Average participant FAS scores per trial without (Phase A) and with (Phase B) COACH

**Participant [MMSE]**	Mean FAS* in Phase A	Mean FAS* in Phase B	**Change (%)**
S1 [4]	4.95	14.14	186
S3 [15]	34.89	35.00	0
S4 [12]	32.00	34.60	8
S5 [20]	31.50	32.43	3
S6 [13]	34.90	34.89	0
S8 [11]	33.00	32.71	-1

* Functional assessment score

### Device performance

A summary of the data regarding COACH performance is presented in Table 9. It should be noted that the item *Participant ignored prompt from COACH *in Table 9 represents the combined number of both ignored hits and ignored false alarms. The error rates, E_w _and E_c _(described by Equations 1 and 2), were found to be 10.9% and 26.0% respectively. This can be interpreted as COACH not responding to 10.9% of the errors made by participants (E_w_) and COACH making an error in 26% of the cases where the participant was completing the step correctly (E_c_).

**Table 9:** Device performance with regards to COACH's response to participants' actions and participants' reactions to prompts given by COACH.

Opportunity outcome*	**Water On**	**Use Soap**	**Rinse Soap**	**Water Off**	**Use Towel**	**TOTAL**
Hit	16	62	15	48	6	147
Miss	1	11	1	3	2	18
False alarm	7	107	18	18	2	152
Correct reject	117	69	87	60	100	433
Steps completed correctly in response to a prompt from COACH	1	4	6	18	1	30
Prompts from the COACH ignored by participants	20	159	24	43	7	253

*Hits, misses, false alarms and correct rejects are defined in Table 3.

## Discussion

### Inter-rater agreement

Altman [35] has indicated that values of K can be interpreted as very good agreement if they are between 0.81 and 1.00 and as good agreement if they are between 0.61 and 0.80. While the K-value for independent step completion and the p-values obtained for all three measures were good, the K-values for the observed number of caregiver interactions (K = 0.69) and FAS (K = 0.63) were at the lower end of the agreement range. Although both raters had previous experience and agreed on scoring conventions, many instances were more difficult to score than one might imagine. For example, 'guidance' was considered to be a prompt and could be a verbal cue, visual cue, tactile cue or any combination of the three. When combined, prompts were difficult to clearly distinguish, such as when verbal guidance was followed closely by visual guidance. In this particular example, it could be interpreted as a single prompt (a verbal plus visual cue) by one rater and as two (first a verbal prompt, followed by a separate visual prompt) by the second rater. Similar scoring difficulties were encountered with the FAS, where the ratings one through four represent the rater's opinion of how much of the step (0 to 25%, 25 to 50%, 50 to 75%, 75 to 100%) the participant completed independently after being given a prompt by the caregiver.

### Participant performance

The participants showed a general improvement in the handwashing task when COACH was used, reflected in more steps completed independently, fewer interactions with the caregiver, and higher FASs. Results presented in Table 6 suggest that improvements in handwashing independence when COACH is used are inversely proportional to a person's baseline performance, with greater levels of improvement being seen by those who are less independent when washing his/her hands. Moreover, four moderately impaired participants who routinely required some assistance (S3, S4, S6, and S8) competed all five handwashing steps completely independently the majority of the time COACH was used. It appears that the improvement is a result of device use rather than activity learning by the participants, supported by the fact that performance improved when COACH was used in B_1_, fell when it was removed in the A_2 _phase, and was regained in B_2 _when COACH was reintroduced (as seen in Table 5). There was a noticeable trend towards a decrease in the number of interactions with the caregiver during the intervention phases, with three of the subjects (S3, S4 and S6) requiring no human assistance when COACH was used. S5 also showed a considerable decrease, requiring only a third of the original number of interactions to complete handwashing. The change in the amount of assistance the participants required was mildly reflected by their FASs (presented in Table 8), although the change is not as obvious as it is with independence and interaction with caregiver measures (Tables 5 and 6). S4's independence from a human caregiver when COACH is shown by her increase to a near perfect FAS. The reason S4 did not achieve a perfect score is because she was a relatively slow hand washer and the FAS dictates that a score of 6 is assigned when the participant "took more than reasonable time to complete step". Participants S3 and S6 were able to perform most of the steps in handwashing independently before the introduction of COACH, therefore these participants had no change in their FAS because there was little opportunity for them to improve.

S8 was a noteworthy subject as she was highly independent, but would consistently omit the soap application step. Although COACH provided a prompt for her to do so at almost every instance, she ignored these prompts and would only respond to verbal prompts from a human caregiver. Thus, essentially no change was seen in S8's number of caregiver interactions or FAS. S8 provides a good example of a user (in terms of ADL and cognitive abilities) who may not be a good candidate for this style of computer-based guidance as her idiosyncrasies resulted in compliance with verbal cues only when they are given by a human.

For the more independent participants (S3 and S6) COACH appears to function more as a "maintenance" tool, able to support the participant in the occurrence of an occasional error. S4, who needed modest amounts of mostly verbal assistance from her caregiver, became essentially independent when COACH was in use. Results from trials involving S3 and S6 suggest that using COACH does not have a detrimental effect on the performance of capable individuals. This indicates that the device could potentially be introduced in the early stages of dementia without any negative effects. An early introduction would allow the system to learn about the user's preferred handwashing regime, which in turn could enable the system to make better decisions when guiding the individual through the activity later on when s/he does require assistance. From this study, it appears that by providing prompts to users with moderate-level dementia, COACH successfully encouraged more independent behavior with greater relative results seen in individuals who required higher levels of assistance to complete the task.

S1 was an exceptional subject in the study. She had the lowest MMSE score and was the only participant who was classified as having severe dementia. Moreover, she showed a notable decline in general abilities over the course of the study, which is reflected in her low and decreasing MMSE scores (Table 4). Although there is not enough data to support any significant conclusions, S1's decline may explain why there is a slight increase in the number of caregiver interactions when the device is used (Table 7), even though her ability to complete steps independently improved during the intervention phases (Table 6). It is thought that while COACH helped to remind S1 which step came next (i.e., improving her independence), she required more and more prompting as the study progressed and her dementia became more severe (i.e., increasing number of caregiver interactions). This supposition is supported by the prompting behavior exhibited by COACH. In B_1_, S1 completed a correct step as a result of five of COACH's 33 correct prompts while in B_2 _S1 did not complete any steps in response to COACH's 40 correct prompts. S1 had 10 trials that required an exceptionally high (for this sample group) 20 or more interactions with the caregiver to complete the handwashing activity. S1 showed the most relative improvement in self-sufficiency (particularly with rinsing off soap and hand drying) and this is reflected by her FAS. However, dependent people such as S1 may require the device to be a constant presence and they will likely need physical assistance from a caregiver with some steps (in particular, getting soap).

The results presented here support the use of this type of technology to increase user independence while decreasing number of interactions with the caregiver, with four out of five of the moderate-level subjects able to complete the activity without any assistance whatsoever from the caregiver when the device was in use. The authors feel that research questions one and two have been adequately addressed and that the use of COACH results in increased independence from a caregiver as well as a reduction in caregiver burden for people with moderate-level dementia. Participants who had only minor difficulties completing the task were the ones who were most likely to become independent when the device was used. These findings agree with studies conducted with previous versions of the COACH, and suggest that COACH has the potential to increase the independence and autonomy of individuals suffering from dementia. Ideally, a system like COACH will enable caregivers to perform other tasks while the completion of ADL are supervised by the system because the system would bring any difficulties regarding task completion to the attention of the caregiver. Therefore, while use of COACH would not eliminate the need for a caregiver (as the caregiver would have to still be present in the home to provide assistance that is beyond the capabilities of a computerised reminder system), it could potentially augment the burden of constant supervision of his/her loved one. This would allow more free time for the caregiver and more privacy for the person with dementia, which in turn would hopefully improve quality of life for the dyad and delay long-term care placement.

### Device performance

**Figure 7:**
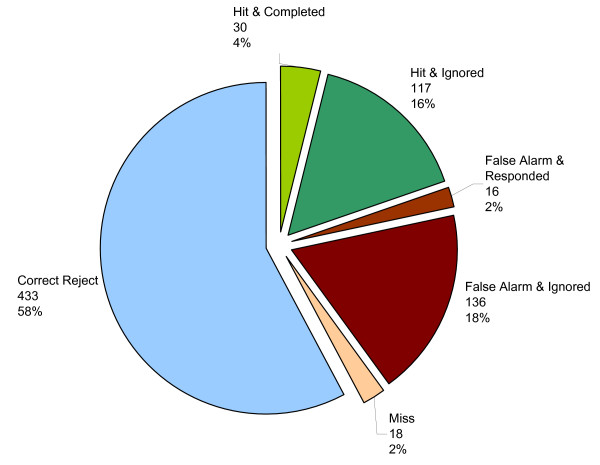
**Observed COACH conditions with corresponding number of observations and percentage of the all observed conditions**. Note that correct prompts (hits) and incorrect prompts (false alarms) are separated into the two observed participant reactions to the prompts: *completed/responded or ignored*.

As device performance examines the interactions between the device, the user, and their environment, the data from all six participants has been grouped and is presented in the discussion below (as opposed to the participants' performance data that were separated according to cognitive impairment). The overall efficacy of the COACH system and its functioning as an assistive technology can be measured primarily by the ability of the COACH to identify an error by the participant and provide correct assistance in response. During this study, COACH had a total of 750 observed conditions, which are summarized in Table 9 and Figure 7. Of these, 170 (23%) were errors (i.e., a false alarm or a miss, as defined in Table 3). Misses and false alarms by the COACH occurred because the system misinterpreted a step in the handwashing task. For example, an ambiguous user action such as touching the taps may have caused the system to incorrectly presume that the water had been turned on. Wrong assumptions by COACH reduce the probability that the correct course of action will be taken. If COACH is not able to correct itself through other observations, a prompt may then be either missed or provided for the wrong step.

From a clinical point of view, the impact of the device's errors on participant performance should be used to determine if the device error rate was acceptable. False alarms sometimes irritated and confused the participants, and it was observed that participants who were confused or frustrated were less likely to complete the remainder of the task if subsequent correct cues were given. Three participants verbally responded to the device when a false alarm was given, expressing that they had already completed the step they were being prompting to do. For example, when prompted incorrectly by COACH, S6 would often ask "What [did] I do?" then in frustration would tell the COACH to "Shut up". When misses occurred the participant was usually unable to complete the rest of the task on his/her own, even if COACH recovered from the miss and prompted the participant correctly, hence trials where misses occurred often had COACH summoning the caregiver to intervene (which was the correct action to be taken by the system by this time). Although this resulted in an increase in the amount of interactions between the participant and the caregiver and a decrease in the number of steps the participant was able complete on their own, this tended to result in less frustration for the participant than false alarms. Thus while misses and false alarms do not seem to cause significant upset in the participants, it is very likely the irritation and confusion resulting from mistakes by COACH hindered participant performance. Reducing the number of these types of system errors is the primary priority of future work on the COACH.

This study uncovered sequences and scenarios that led to false alarm prompts in this particular COACH model. Analysis of the data revealed that 107 (70%) of all false alarm prompts issued were to "use the soap", equating to 36% of all prompts given by the device or 8% of all observed conditions. Clearly, incorrect prompting for soap use constitutes the majority of the errors committed by COACH. Further investigation of sequences where "use the soap" false alarm errors occurred revealed that 59 (55%) of the "use the soap" false alarms took place after the completion of the handwashing task (i.e. after all of the five essential steps were completed), mostly when the participant was drying his/her hands. Closer examination of these trials revealed that the tracking, policy and prompting modules appeared to be working well, therefore the authors conclude the problem is likely in the belief state monitor. For many of these trials, when the user reaches the end of the handwashing activity and is drying his/her hands, the COACH correctly shows the activity as being complete. However, after several seconds the belief state monitor slowly changes its belief distribution from a strong belief that handwashing is complete to the belief that the participant's hands are not washed. When the belief that the participant's hands were not washed reached a sufficient level, a prompt to "use the soap" occurs. This "drift" back to the beginning of the task was intentionally designed to give the system an opportunity to correct itself if it incorrectly believed that the user had completed the task. However, the clinical trials showed that the usefulness of this functionality may not outweigh the clinical costs associated with the resulting erroneous prompts, particularly if the participant is someone who takes a long time to dry his/her hands. This problem is being corrected in the next version of COACH by implementing a "cutoff" where the system terminates guidance once the system's belief that the user's hands are clean and dry reaches an empirically determined threshold, at which point the caregiver will be called to escort the participant from the washroom.

As presented in Table 9, COACH had 580 correct conditions (77% of overall conditions), composed of 147 correct prompts (or hits) when participants required assistance and 443 correct rejects, where no prompt was issued because the participant did not need assistance. Of the correct prompts given to participants, 30 (20% of all hits) resulted in the participant complying with the cue and completing the step accordingly (Figure 7). The number of correct prompts ignored by participants ranged dramatically from one (by S6) to 68 (by S1). Notably, the number of correct prompts ignored by S1 (severely-impaired) account for over half (58%) of the total correct prompts ignored by all participants, with only five of the correct prompts given to S1 resulting in completion of the step. In contrast, S4 was the most responsive to hits, completing a step in response to the COACH 60% of time she was provided with support. In the more severe stages of AD individuals tend to lose the ability to respond to his/her environment, the ability to speak and, ultimately, the ability to control movement [36]. This may explain S1's lack of response to the system; however, it does not explain why S5, who has the highest MMSE, had a lower handwashing performance than any of the participants with moderate dementia. S5 responded well to COACH, completing a step in 43% of the cases when a correct prompt (hit) was given.

Sixteen of the 152 false alarms that were given (11% of all false alarms) elicited reactions from the participants (Figure 7). It should be noted that a response to a prompt is not considered to be the same as the completion of a step, as an unsuccessful *attempt *to complete a step is also considered a response. For instance, if COACH (correctly or incorrectly) prompted a participant to "turn the water on" and the participant touched the taps without altering water flow, this would be considered a reaction to the prompt, although no progress was made in the step itself. Therefore, the response rate to false alarms of 11% is considerably lower than the 20% completion rate for correct prompts when considering the compliance rate with prompts in terms of step completion. When participants did perform an incorrect action because of a false alarm from COACH (such as using the soap when the participant's hands were already washed), this often resulted in backtracking in the activity, adding to the number of steps required for activity completion. It was observed that the extra steps and confusion resulting from backtracking offered a greater opportunity for errors to occur. As such, assistance from the caregiver was usually needed to complete the task. At 34%, S5 had the highest response rate to system prompts regardless of whether they were a hit or a false alarm. S5's high compliance rate may explain why his FAS did not reflect his other improvements as much as they did for the other participants in the study (see Tables 5, 6, and 7). When S5 responded to a false alarm, this often led to backtracking in the activity and participant confusion, requiring the caregiver to be summoned, who then had to use greater levels of assistance to re-orient S5, which would result in lower FAS results.

COACH autonomously used observations of each participant's actions and responses to prompts to estimate his/her level of dementia (a parameter that changed slowly over a course of days) and responsiveness (a parameter that changed on a day-to-day basis). These parameters played a large role in dictating the level of detail of the prompts given by COACH, which would select prompts that were appropriate for the individual's levels of responsiveness and dementia. From the technical results presented in Hoey et al. [19], COACH autonomously assigned a *low dementia level *(i.e. more impaired) to all but one participant, S5, who had a rating of *medium level dementia*. It is interesting to note that these ratings coincide with the participant's MMSE scores rather than participant performance during the handwashing activity. These results suggest an interesting possible additional application of COACH as a diagnostic tool; by watching older adults perform ADL over a series of weeks or months, a future version COACH may be used to detect changes in the users' abilities, and consequently, level of dementia. COACH's ordering of the prompts was also appropriate. The majority of instances showed the system prompting the participant using a well-timed progression of prompting strategies from audio (only audio), video (audio and video), and finally to summoning the caregiver to intervene if the participant was unresponsive to the prompts. COACH would autonomously carry estimates about the user's dementia level from one trial to the next, so that the system would not have to relearn participant behaviors for each trial.

Using the participant's name at the beginning of each cue was a successful technique to gain his/her attention. For the majority of the prompts, regardless of whether they were hits or false alarms, the participant would look up at the video screen in anticipation of further instruction when s/he heard his/her name. If a video was played, most participants would watch the video to its completion, regardless of whether or not they responded to the prompt. The wording of the prompts was appropriate for this population as participants often spoke back to the prompts with a reply that showed they understood what was being asked, even if they did not comply. For example, when prompted by COACH to use the soap, S8 would often say "No thank you, I don't want to". As participants usually distinctly looked up at the screen when they heard their name, (when played) watched video cues to completion, and provided coherent responses to prompts, it can be concluded that the audio and audio-video cuing techniques used in this study are successful at getting the attention of most older adults with dementia, although attention span and compliance is dependant on the individual traits of the participant.

COACH waited a pre-determined amount of time before giving a prompt to the participant if s/he was "stuck" on a step, with COACH giving the participant ample time to attempt the step on his/her own before prompting. COACH's "patience" may have played a role in the greater levels of independence seen when the device is used, as participants would sometimes correctly resume the task after a pause of a few seconds; a pause which may be longer than most human caregivers would care to wait. However, COACH would sometimes be too patient. Prompts to correct a participant when they had confused the ordering of steps were often given several seconds after they should have been. There were several instances where the participant had completed at least one, and sometimes several, incorrect step(s) before COACH gave the appropriate corrective prompt. As such, it is quite possible that the participants would ignore fewer prompts from COACH if the timing of the prompts was improved. The authors are implementing the use of automatically determined, participant specific pauses between the delivery of prompts, which will be dynamically adjusted in response to the individual's dementia and responsiveness levels.

While there have been areas of the system identified for improvement, based on these and previous COACH trials, the authors speculate that participant responsiveness is dependant on several traits of the particular individual, such as cognitive abilities, hearing, vision, mood, compliance, and general attitude. Using a POMDP as the planning agent for this model allowed the system to estimate unobservable user traits such as responsiveness and dementia level to help tailor timing and level of prompts to the abilities of the individual, however, the ultimate usefulness of this type of assistive technology is very much dependant on the traits of the person who is using it. It is of significant interest that the level of dementia (as determined by the MMSE) alone does not appear to be an indicator of an individual's success with this type of technology.

### Limitations

There are several limitations regarding this study that must be acknowledged. While the COACH shows promising results, the sample size was too small to draw any significant conclusions about wide-scale applicability or performance. The study presented here focused on a moderate-to-severe level dementia, with the majority of the participants being from the moderate group. Although the researchers believe the trends seen here would also be seen in mild and severe populations, to what extent remains unknown until the device can be tested with a larger group that contains a diversity of dementia levels. More testing with a broader population, including informal caregivers, must be done before any significant conclusions regarding this device can be made.

### Future Work

The results gathered here are convincing enough that the authors are preparing the device for the next set of clinical trials, which will include more participants and will be conducted within homes in the community. Some improvements that the researchers hope to include are the adaptation of the system to distinguish between multiple washroom ADL such as tooth brushing and, eventually, toileting. The team plans to examine the implementation of speech recognition to allow the system to recognize other types of implicit user feedback. Multiple camera input (vision/tracking systems) gained by placing cameras in various positions throughout the washroom could provide greater user observation accuracy and versatility and would enable 3D observations. It is hoped that the next set of trials can be performed over a longer period of time with a larger sample size to decrease the effects of the natural performance variability that is found in this population.

## Conclusion

This paper presents the results from clinical trials with a small group of potential users of the COACH, a cognitive assistive technology designed to assist older adults with moderate to severe dementia through ADL. This study aimed to determine whether or not the POMDP-based COACH system was capable of: 1) reducing user dependence on a caregiver, 2) decreasing caregiver workload, and/or 3) providing correct guidance through the handwashing task. When COACH was used, the participants appeared to show an increase in the number of handwashing steps they were able to complete without assistance from the caregiver as well as the decrease in number of times they required assistance from the caregiver during the activity. Four of the five moderate-level participants were independent from a human caregiver during handwashing when COACH was used. Based on these results, this study has affirmatively answered the first two research questions. Through these clinical trials, the POMDP-based planning system shows promise as a possible planning algorithm for guiding older adults with dementia through handwashing, albeit several areas in need of improvement have been identified. These improvements will be made and tested before the next set of clinical trials begin, which are planned to be supervised community-based (as opposed to long-term care facility-based) trials starting in 2009. It is hoped that the next set of trials will allow the authors to answer these research questions more definitively with a lager sample size that includes a greater diversity in dementia levels.

In general the participants were less dependent on a human caregiver when COACH was used. As the effectiveness varied considerably and seemed to be dependent on each individual's idiosyncrasies, these findings suggest that COACH could be useful to the caregiving dyads of individuals who respond well to prompting without tactile cues. These findings also support the importance of understanding the special, diverse and dynamic needs of this target user group to ensure that appropriate, customizable assistance is available in assistive technologies to help support people with dementia and their caregivers.

## Abbreviations

AD: Alzheimer's disease; ADL: Activity of daily living; AI: Artificial Intelligence; CAT: Cognitive assistive orthosis; COACH: **C**ognitive **O**rthosis for **A**ssisting with a**C**tivities in the **H**ome; FAS: Functional assessment score; MMSE: Mini Mental State Examination.

## Competing interests

The authors declare that they have no competing interests.

## Authors' contributions

AM supervised the project, developed the study design, and assisted in the preparation and editing of the manuscript. JB participated in the study design, test area set-up, second rating of the trials, and drafting the manuscript. TC assisted in running trials, was the primary rater, and participated in drafting the manuscript. JH designed the software algorithms used in this study. All authors participated in the preparation of the final manuscript.

## Pre-publication history

The pre-publication history for this paper can be accessed here:


